# Obesity and kidney disease: hidden consequences of the epidemic

**DOI:** 10.1590/1414-431X20166075

**Published:** 2017-04-13

**Authors:** C.P. Kovesdy, S.L. Furth, C. Zoccali

**Affiliations:** 1 Division of Nephrology, Department of Medicine, University of Tennessee Health Science Center, Memphis, TN, USA; 2 Nephrology Section, Memphis VA Medical Center, Memphis, TN, USA; 3 Department of Pediatrics, Perelman School of Medicine, University of Pennsylvania, Philadelphia, PA, USA; 4 CNR-IFC Clinical Epidemiology and Pathophysiology of Renal Diseases and Hypertension, Reggio Calabria, Italy

**Keywords:** Obesity, Chronic kidney disease, Nephrolithiasis, Kidney cancer, Prevention

## Abstract

Obesity has become a worldwide epidemic and its prevalence has been projected to grow by 40% in the next decade. This increasing prevalence has implications for the risk of diabetes, cardiovascular disease and also for chronic kidney disease (CKD). A high body mass index is one of the strongest risk factors for new-onset CKD. In individuals affected by obesity, a compensatory hyperfiltration occurs to meet the heightened metabolic demands of the increased body weight. The increase in intraglomerular pressure can damage the kidneys and raise the risk of developing CKD in the long-term. The incidence of obesity-related glomerulopathy has increased ten-fold in recent years. Obesity has also been shown to be a risk factor for nephrolithiasis, and for a number of malignancies including kidney cancer. This year, the World Kidney Day will promote education on the harmful consequences of obesity and its association with kidney disease, advocating healthy lifestyle and health policy measures that make preventive behaviors an affordable option.

## Introduction

In 2014, over 600 million adults (18 years and older) worldwide were obese. Obesity is a potent risk factor for the development of kidney disease. It increases the risk of developing major risk factors for chronic kidney disease (CKD), like diabetes and hypertension, and it has a direct impact on the development of CKD and end-stage renal disease (ESRD). In individuals affected by obesity, a compensatory mechanism of hyperfiltration likely occurs to meet the heightened metabolic demands of the increased body weight. The increase in intraglomerular pressure can damage the kidney structure and raise the risk of developing CKD in the long-term.

The good news is that obesity, as well as the related CKD, are largely preventable. Education and awareness of the risks of obesity and a healthy lifestyle, including proper nutrition and exercise, can dramatically help in preventing obesity and kidney disease. This article reviews the association of obesity with kidney disease on the occasion of the 2017 World Kidney Day.

## Epidemiology of obesity in adults and children

Over the last 3 decades, the prevalence of overweight and obese adults (BMI ≥25 kg/m^2^) worldwide has increased substantially (1). In the USA, the age-adjusted prevalence of obesity in 2013-2014 was 35% among men and 40.4% among women (2). The problem of obesity also affects children. In the USA in 2011-2014, the prevalence of obesity was 17% and extreme obesity 5.8% among youth 2-19 years of age. The rise in obesity prevalence is also a worldwide concern (3,4), as it is projected to grow by 40% across the globe in the next decade. Low- and middle-income countries are now showing evidence of transitioning from normal weight to overweight and obesity as parts of Europe and the United States did decades ago (5). This increasing prevalence of obesity has implications for cardiovascular disease (CVD) and also for CKD. A high body mass index (BMI) is one of the strongest risk factors for new-onset CKD (6,7).

Definitions of obesity are most often based on BMI [i.e., weight (kilograms) divided by the square height (meters)]. A BMI between 18.5 and 25 kg/m^2^ is considered by the World Health Organization (WHO) to be normal weight, a BMI between 25 and 30 kg/m^2^ as overweight, and a BMI of >30 kg/m^2^ as obese. Although BMI is easy to calculate, it is a poor estimate of fat mass distribution, as muscular individuals or those with more subcutaneous fat may have a BMI as high as individuals with larger intra-abdominal (visceral) fat. The latter type of high BMI is associated with substantially higher risk of metabolic and cardiovascular disease. Alternative parameters to more accurately capture visceral fat include waist circumference (WC) and a waist hip ratio (WHR) of >102 and 0.9 cm, respectively, for men and >88 and >0.8 cm, respectively, for women. WHR has been shown to be superior to BMI for the correct classification of obesity in CKD.

## Association of obesity with CKD and other renal complications

Numerous population-based studies have shown an association between measures of obesity and both the development and the progression of CKD (Table 1). Higher BMI is associated with the presence (8) and development (9–11) of proteinuria in individuals without kidney disease. Furthermore, in numerous large population-based studies, higher BMI appears associated with the presence (8,12) and development of low estimated glomerular filtration rate (GFR) (9,10,13), with more rapid loss of estimated GFR over time (14), and with the incidence of ESRD (15–18). Elevated BMI levels, class II obesity and above, have been associated with more rapid progression of CKD in patients with pre-existing CKD (19). A few studies examining the association of abdominal obesity using WHR or WC with CKD, describe an association between higher girth and albuminuria (20), decreased GFR (8) or incident ESRD (21) independent of BMI level.

**Table 1:**
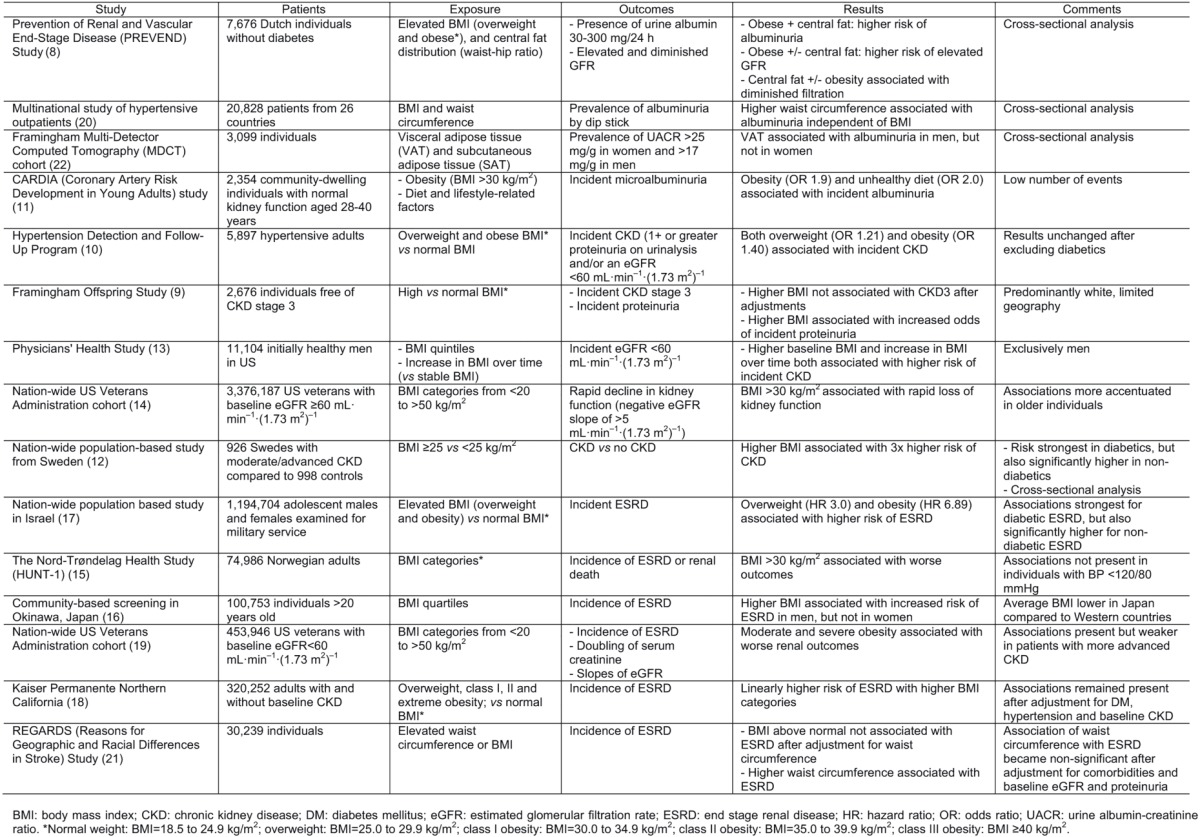
Stides examining the association of obesity with various measures of chronic kidney disease.

Higher visceral adipose tissue measured by computed tomography has been associated with a higher prevalence of albuminuria in men (22). The observation of a BMI-independent association between abdominal obesity and poorer renal outcomes is also described in relationship with mortality in patients with ESRD (23) and kidney transplant (24), and suggests a direct role of visceral adiposity. In general, the associations between obesity and poorer renal outcomes persist even after adjustments for possible mediators of obesity’s cardiovascular and metabolic effects, such as high blood pressure and diabetes mellitus, suggesting that obesity may affect kidney function through mechanisms in part unrelated to these complications (see next section).

The deleterious effect of obesity on the kidneys extends to other complications such as nephrolithiasis and kidney malignancies. Higher BMI is associated with an increased prevalence (25) and incidence (26,27) of nephrolithiasis. Furthermore, weight gain over time and higher baseline WC were also associated with higher incidence of nephrolithiasis (27). Obesity is associated with various types of malignancies, particularly cancers of the kidneys. In a population-based study of 5.24 million individuals from the UK, a 5-kg/m^2^ higher BMI was associated with a 25% higher risk of kidney cancers, with 10% of all kidney cancers attributable to excess weight (28). Another large analysis examining the global burden of obesity on malignancies estimated that 17 and 26% of all kidney cancers in men and women, respectively, were attributable to excess weight (29). The association between obesity and kidney cancers was consistent in both men and women, and across populations from different parts of the world in a meta-analysis that included data from 221 studies (of which 17 examined kidney cancers). Among the cancers examined in this meta-analysis, kidney cancers had the third highest risk associated with obesity (relative risk per 5 kg/m^2^ higher BMI=1.24, 95%CI=1.20-1.28, P<0.0001) (30).

## Mechanisms of action underlying the renal effects of obesity

**Figure 1:**
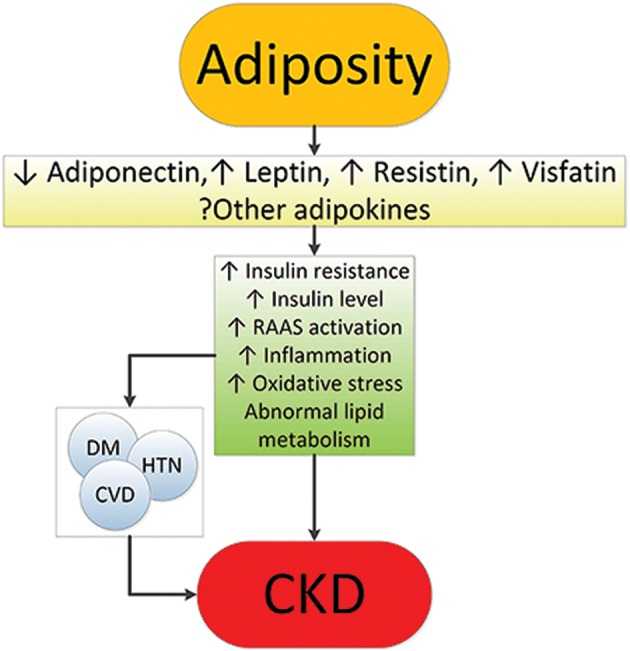
Putative mechanisms of action whereby obesity causes chronic kidney disease (CKD). RAAS: renin-angiotensin-aldosterone system; DM: diabetes mellitus; HTN: hypertension; CVD: cardiovascular disease.

Obesity results in complex metabolic abnormalities, which have wide-ranging effects on diseases affecting the kidneys. The exact mechanisms whereby obesity may worsen or cause CKD remain unclear. The fact that most obese individuals never develop CKD, and the distinction of as many as 25% of obese individuals as “metabolically healthy” suggests that increased weight alone is not sufficient to induce kidney damage (31). Some of the deleterious renal consequences of obesity may be mediated by downstream comorbid conditions such as diabetes mellitus or hypertension, but there are also effects of adiposity that could impact the kidneys directly induced by the endocrine activity of the adipose tissue via production of adiponectin (32), leptin (33) and resistin (34), among others (Figure 1). These include the development of inflammation (35), oxidative stress (36), abnormal lipid metabolism (37), activation of the renin-angiotensin-aldosterone system (38), and increased production of insulin and insulin resistance (39,40).

**Figure 2:**
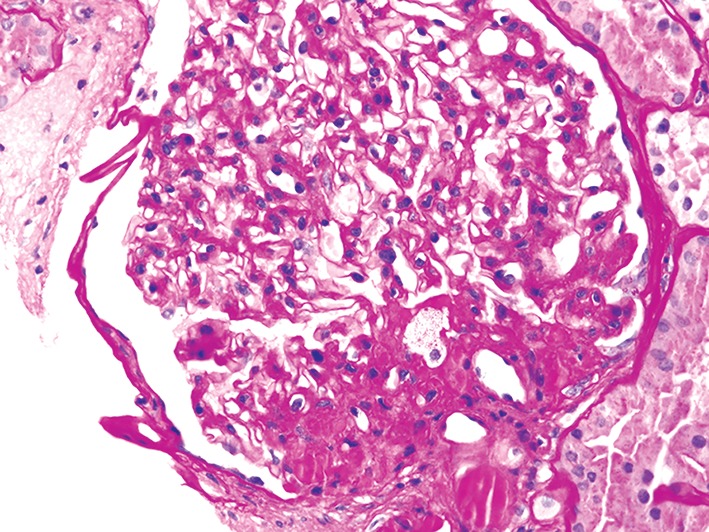
Obesity-related perihilar focal segmental glomerulosclerosis on a background of glomerulomegaly. Periodic Acid-Schiff stain, original magnification 400×. Courtesy of Dr. Patrick D. Walker, MD (Arkana Laboratories, Little Rock, AR, USA).

These various effects result in specific pathological changes in the kidneys (41), which could underlie the higher risk of CKD seen in observational studies. These include ectopic lipid accumulation (42) and increased deposition of renal sinus fat (43,44), the development of glomerular hypertension and increased glomerular permeability caused by hyperfiltration-related glomerular filtration barrier injury (45), and ultimately the development of glomerulomegaly (46), and focal or segmental glomerulosclerosis (41) (Figure 2). The incidence of the so-called obesity-related glomerulopathy (ORG) has increased ten-fold between 1986 and 2000 (41). Importantly, ORG often presents along with pathophysiological processes related to other conditions or advanced age, conspiring to result in more accentuated kidney damage in patients with high blood pressure (47) or in the elderly (14,39).

Obesity is associated with a number of risk factors contributing to the higher incidence and prevalence of nephrolithiasis. Higher body weight is associated with lower urine pH (48 [Bibr B49]) and increased urinary oxalate 49), uric acid, sodium and phosphate excretion (50). Diets richer in protein and sodium may lead to a more acidic urine and a decrease in urinary citrate, also contributing to kidney stone risk. The insulin resistance characteristic of obesity may also predispose to nephrolithiasis (51) through its impact on tubular Na-H exchanger (52) and ammoniagenesis (53), and the promotion of an acidic milieu (54). Complicating the picture is the fact that some weight loss therapies result in a worsening, rather than an improvement in the risk for kidney stone formation; e.g. gastric surgery can lead to a substantial increase in enteral oxalate absorption and enhanced risk of nephrolithiasis (55).

The mechanisms behind the increased risk of kidney cancers observed in obese individuals are less well characterized. Insulin resistance, and the consequent chronic hyperinsulinemia and increased production of insulin-like growth factor 1 and of numerous complex secondary humoral effects may exert stimulating effects on the growth of various types of tumor cells (56). More recently, the endocrine functions of adipose tissue (57), its effects on immunity (58), and the generation of an inflammatory milieu with complex effects on cancers (59,60) have emerged as additional explanations.

## Obesity in patients with advanced kidney disease: The need for a nuanced approach

Considering the above evidence about the overwhelmingly deleterious effects of obesity on various disease processes, it is seemingly counterintuitive that obesity has been consistently associated with lower mortality rates in patients with advanced CKD (19,61) and ESRD (62,63). Similar “paradoxical” associations have also been described in other populations, such as in patients with congestive heart failure (64), chronic obstructive pulmonary disease (65), rheumatoid arthritis (66), and even in old individuals (67). It is possible that the seemingly protective effect of a high BMI is the result of the imperfection of BMI as a measure of obesity, as it does not differentiate the effects of adiposity from those of higher non-adipose tissue. Indeed, studies that separated the effects of a higher WC from those of higher BMI showed a reversal of the negative association with mortality (23,24). Higher muscle mass has also been shown to explain at least some of the positive effects attributed to elevated BMI (63,68). However, there is also evidence to suggest that higher adiposity, especially subcutaneous (non-visceral) fat, may also be associated with better outcomes in ESRD patients (62). Such benefits may indeed be present in patients who have very low short-term life expectancy, such as most ESRD patients (69). Indeed, some studies that examined the association of BMI with time-dependent survival in ESRD have shown a marked contrast between protective short-term effects *vs* deleterious longer-term effects of higher BMI (70). There are several putative short-term benefits that higher body mass could portend, especially to sicker individuals. These include a benefit from the better nutritional status typically seen in obese individuals, and which provides better protein and energy reserves in the face of acute illness, and a higher muscle mass with enhanced antioxidant capacity (63) and lower circulating actin and higher plasma gelsolin levels (71), which are associated with better outcomes. Other hypothetically beneficial characteristics of obesity include a more stable hemodynamic status with mitigation of stress responses and heightened sympathetic and renin-angiotensin activity (72); increased production of adiponectins (73) and soluble tumor necrosis factor alpha receptors (74) by adipose tissue neutralizing the adverse effects of tumor necrosis factor alpha; enhanced binding of circulating endotoxins (75) by the characteristically higher cholesterol levels seen in obesity; and sequestration of uremic toxins by adipose tissue (76).

## Potential interventions for management of obesity

Obesity engenders kidney injury via direct mechanisms through deranged synthesis of various adipose tissue cytokines with nephrotoxic potential, as well as indirectly by triggering diabetes and hypertension, i.e., two conditions that rank among the strongest risk factors for CKD. Perhaps due to the survival advantage of obesity in CKD, the prevalence of end stage kidney disease is on the rise both in the USA (77) and in Europe (78). Strategies for controlling the obesity-related CKD epidemic at population level and for countering the evolution of CKD toward kidney failure in obese patients represent the most tantalizing task that today’s health planners, health managers and nephrologists face.

## Countering CKD at population level

Calls for public health interventions in the community to prevent and treat CKD at an early stage have been made by major renal associations, including the International Society of Nephrology, International Federation of the Kidney Foundation, the European Renal Association, and various national societies. In the USA, Healthy People 2020, a program that sets 10-year health targets for health promotion and prevention, focuses both on CKD and obesity. Surveys to detect obese patients, particularly those with a high risk of CKD (e.g., hypertensive and/or diabetic obese people) and those receiving suboptimal care, to inform these patients of the potential risk for CKD to which they are exposed, is the first step towards developing public health interventions. Acquiring evidence showing that current interventions to reduce CKD risk in the obese are efficacious and deployable is an urgent priority to set goals and means for risk modification. Appropriate documentation of existing knowledge distilling the risk and the benefits of primary and secondary prevention interventions in obese people, and new trials in this population to fill knowledge gaps (see below) are needed. Finally, surveillance programs that monitor progress on the detection of at-risk individuals and the effectiveness of prevention programs being deployed (79) constitute the third, fundamental element for establishing efficacious CKD prevention plans at population level.

A successful surveillance system for CKD has already been implemented in some places such as the UK (80). A campaign to disseminate and apply the Kidney Disease Outcomes Quality Initiative (K-DOQI) CKD guidelines in primary care within the UK National Health Service was launched. This progressively increased the adoption of K-DOQI guidelines and, also thanks to specific incentives for UK general physicians to detect CKD, led to an impressive improvement in the detection and care of CKD, i.e., better control of hypertension and increased use of angiotensin-converting enzyme and angiotensin receptor blockers (80). This system may serve as a platform to improve the prevention of obesity-related CKD. Campaigns aiming at reducing the obesity burden are now at center stage worldwide and are strongly recommended by the WHO, and it is expected that these campaigns will reduce the incidence of obesity-related complications, including CKD. However, obesity-related goals in obese CKD patients remain vaguely formulated, largely because of the paucity of high-level evidence studies to modify obesity in CKD patients (81).

## Prevention of CKD progression in obese people with CKD

Observational studies in metabolically healthy obese subjects show that the obese phenotype unassociated with metabolic abnormalities *per se* predicts a higher risk for incident CKD (82), suggesting that obesity *per se* may engender renal dysfunction and kidney damage even without diabetes or hypertension (see above). In overweight or obese diabetic patients, a lifestyle intervention including caloric restriction and increased physical activity reduced the risk for incident CKD by 30%, compared to a standard follow-up based on education and support to sustain diabetes treatment; although it did not affect the incidence of cardiovascular events (83). Such a protective effect was partly due to reductions in body weight, HbA1c, and systolic blood pressure. No safety concerns regarding kidney-related adverse events were seen (83). In a recent meta-analysis comparing experimental studies in obese CKD patients, interventions aimed at reducing body weight showed coherent reductions in blood pressure, glomerular hyper-filtration and proteinuria (81). A thorough *post-hoc* analysis of the REIN (ramipril in non-diabetic renal failure) study showed that the nephron-protective effect of angiotensin-converting enzymes (ACE) inhibition in proteinuric CKD patients was maximal in obese CKD patients, but minimal in CKD patients with normal or low BMI (84). Of note, bariatric surgical intervention has been suggested for selected CKD and ESRD patients including dialysis patients who are wait-listed for kidney transplantation (85–87).

Globally, these experimental findings provide a proof of concept for the usefulness of weight reduction and ACE inhibition interventions in the treatment of CKD in the obese. Studies showing a survival benefit of increased BMI in CKD patients, however, remain to be explained (88). These findings limit our ability to make strong recommendations about the usefulness and the safety of weight reduction among individuals with more advanced stages of CKD. Lifestyle recommendations to reduce body weight in obese people at risk for CKD and in those with early CKD appear justified, particularly for the control of diabetes and hypertension. As the independent effect of obesity control on the incidence and progression of CKD is difficult to disentangle from the effects of hypertension and type 2 diabetes, recommendation of weight loss for the minority of metabolically healthy, non-hypertensive obese patients remains unwarranted. These considerations suggest that a therapeutic approach to overweight and obesity in patients with advanced CKD or other significant comorbid conditions has to be pursued carefully, with proper considerations of the expected benefits and potential complications of weight loss over the life span of the individual patient.

## Conclusions

The worldwide epidemic of obesity affects the Earth’s population in many ways. Diseases of the kidneys, including CKD, nephrolithiasis and kidney cancers are among the more insidious effects of obesity, but which nonetheless have wide ranging deleterious consequences, ultimately leading to significant excess morbidity and mortality and excess costs to individuals and the entire society. Population-wide interventions to control obesity could have beneficial effects in preventing the development or delaying the progression of CKD. It is incumbent upon the entire healthcare community to devise long-ranging strategies towards improving the understanding of the links between obesity and kidney diseases, and to determine optimal strategies to stem the tide. The 2017 World Kidney Day is an important opportunity to increase education and awareness to that end.
